# ATRX histone binding and helicase activities have distinct roles in neuronal differentiation

**DOI:** 10.1093/nar/gkac683

**Published:** 2022-08-24

**Authors:** Anna Bieluszewska, Phillip Wulfridge, John Doherty, Wenqing Ren, Kavitha Sarma

**Affiliations:** Gene Expression and Regulation Program, The Wistar Institute, Philadelphia, PA 19104, USA; Epigenetics Institute, University of Pennsylvania, Philadelphia, PA 19104, USA; Gene Expression and Regulation Program, The Wistar Institute, Philadelphia, PA 19104, USA; Epigenetics Institute, University of Pennsylvania, Philadelphia, PA 19104, USA; Gene Expression and Regulation Program, The Wistar Institute, Philadelphia, PA 19104, USA; Epigenetics Institute, University of Pennsylvania, Philadelphia, PA 19104, USA; Gene Expression and Regulation Program, The Wistar Institute, Philadelphia, PA 19104, USA; Epigenetics Institute, University of Pennsylvania, Philadelphia, PA 19104, USA; Gene Expression and Regulation Program, The Wistar Institute, Philadelphia, PA 19104, USA; Epigenetics Institute, University of Pennsylvania, Philadelphia, PA 19104, USA

## Abstract

ATRX is a chromatin remodeler, which is mutated in ATRX syndrome, a neurodevelopmental disorder. ATRX mutations that alter histone binding or chromatin remodeling activities cluster in the PHD finger or the helicase domain respectively. Using engineered mouse embryonic stem cells that exclusively express ATRX protein with mutations in the PHD finger (PHDmut) or helicase domains (K1584R), we examine how specific ATRX mutations affect neurodifferentiation. ATRX PHDmut and K1584R proteins interact with the DAXX histone chaperone but show reduced localization to pericentromeres. Neurodifferentiation is both delayed and compromised in PHDmut and K1584R, and manifest differently from complete ATRX loss. We observe reduced enrichment of PHDmut protein to ATRX targets, while K1584R accumulates at these sites. Interestingly, ATRX mutations have distinct effects on the genome-wide localization of the polycomb repressive complex 2 (PRC2), with PHDmut and ATRX knockout showing reduced PRC2 binding at polycomb targets and K1584R showing loss at some sites and gains at others. Notably, each mutation associated with unique gene signatures, suggesting distinct pathways leading to impaired neurodifferentiation. Our results indicate that the histone binding and chromatin remodeling functions of ATRX play non-redundant roles in neurodevelopment, and when mutated lead to ATRX syndrome through separate regulatory pathways.

## INTRODUCTION

Chromatin regulators are frequently mutated in several neurodevelopmental disorders. Mutations in the ATRX chromatin remodeler cause a severe neurodevelopmental disorder known as ATRX syndrome (1). ATRX has well-established roles in important developmental processes, including histone H3.3 deposition as part of the ATRX/DAXX complex (2–5); silencing of repetitive elements to maintain genome stability (6,7), and X chromosome inactivation (8,9). ATRX loss is associated with accumulation of G-quadruplex (G4) (10,11) and R-loop structures at repetitive regions such as telomeres (12,13) and increased replicative stress (10,14). ATRX also interacts with and regulates the localization of the methyl CpG binding protein MeCP2 (15,16). ATRX also facilitates the localization of the polycomb repressive complex 2 (PRC2) to the inactive X chromosome and to polycomb targets genome-wide through an unknown mechanism (8).

ATRX is a large 280 kDa protein with distinct domains that mediate its interactions with chromatin. ATRX contains an atypical plant homeodomain (PHD) finger domain that recognizes a combinatorial modification pattern on histone H3 tails. Structural studies show that the ATRX PHD finger binds histone H3 tails that are unmodified at lysine 4 (H3K4me0) and trimethylated at lysine 9 (H3K9me3) (17–20). ATRX also contains a helicase domain through which it interacts with DNA to remodel nucleosomes (5). ATRX helicase activity was also shown to resolve DNA triplex structures *in vitro* (21,22). Both the PHD finger and helicase domains are highly conserved across species. ATRX is also a high affinity RNA binding protein that interacts with the Xist long non-coding RNA, the master regulator of dosage compensation in mammals (8). The non-canonical RNA binding region of ATRX is distinct from its histone and DNA binding domains (23). Recent biochemical studies indicate that the RNA binding property of ATRX prevents the assembly of R-loop structures *in vitro* (24). Interestingly, ATRX is unable to resolve both R-loop (24) and DNA G4 (14) structures *in vitro*. Thus, through its distinct chromatin interaction domains, ATRX plays an essential role in regulating gene expression and genome stability during development.

The majority of ATRX syndrome mutations cluster in the PHD finger (∼50%) and helicase domains (∼30%). Most ATRX syndrome mutations result in protein instability and reduced overall levels of ATRX protein (21). However, the developmental consequences of PHD finger and helicase domain mutations are distinct. Mutations in the PHD finger are associated with severe intellectual disability and psychomotor impairment, while mutations in the helicase domain often manifest with milder neurodevelopmental delays but more severe genital abnormalities (25). This genotype-phenotype correlation suggests a more complex mechanism for disease than ATRX haploinsufficiency alone. The distinct and non-redundant functions of the PHD and helicase domains are also apparent from studies in *Drosophila*, which contains two orthologues to the *ATRX* gene: *dxnp* and *dadd1*. *Drosophila* ADD1 and XNP proteins are the ATRX orthologs of the human ADD and SNF2 domains, respectively. Analyses of retrotransposons in mutant strains indicate that dXNP and dAdd1 suppress transcription from retrotransposon elements, but dXNP specifically prevents retrotransposon integration (26). Similarly, both dXNP and dAdd1 regulate Heterochromatin protein 1a (HP1a) localization to telomeres. Loss of dAdd1 uniformly affected HP1a enrichment at all telomeres, while dXNP loss had impacts only at some telomeres.

A clearer understanding of pathways regulated by the PHD finger or helicase domains may provide a basis for mutation specific differences during development. Here, using a mouse embryonic stem cell (mESC) model where we exclusively express separation-of-function ATRX PHD finger or helicase domain mutants, we examine the impact of ATRX mutations on neurodifferentiation. We identify specific effects on ATRX and polycomb protein localization and uniquely altered gene expression signatures that can affect neurodevelopment.

## MATERIALS AND METHODS

### Cell lines and cell culture

E14 mouse embryonic stem cells were cultured as described previously (24,27). Cells were grown on 0.1% gelatin coated plates in media containing DMEM, 15% fetal bovine serum (Gibco), 1× MEM non-essential amino acids, 1× GlutaMAX (Gibco 35050), 25 mM HEPES, 100 U/ml Pen-Strep and 55 μM 2-mercaptoethanol, 3 μM glycogen synthase kinase (GSK) inhibitor (Millipore 361559), 1 μM MEK1/2 inhibitor (Millipore 444966) and LIF (Sigma, ESGRO).

Differentiation of mESCs to NPCs was performed as previously described (24). mESCs were plated into gelatin-coated wells of a six-well plate (30 000 cells per well) in mESC medium (see Cell culture) and cultured overnight to allow attachment to the plate. To induce differentiation, mESC medium was withdrawn and N2B27 medium (50% Neurobasal medium, 50% DMEM/F-12 medium, 1 mM sodium pyruvate, 0.1 mM non-essential amino acids, 2 mM l-glutamine, 0.5% Pen-Strep, 55 μM beta-mercaptoethanol, 40 μG/ml bovine serum albumin, 1× N-2 supplement, 1× B-227 supplement) containing 10 ng/ml human basic fibroblast growth factor (bFGF, Gemini Bio #300-112P) was added. Media was replaced with N2B27 medium containing bFGF at 24 and 48 h after induction. At 72 and 96 h after induction, media was replaced with N2B27 medium containing 500 nM smoothened agonist (SAG, Sigma #566661). Cells were imaged at 72 (day 3) and 144 h (day 6) after induction.

### Generation of cell lines

Guide RNAs were designed using the CRISPR Design Tool (https://zlab.bio/guide-design-resources) and inserted into PX459, a gift from Feng Zhang (Addgene plasmid: 62988) (28). Gene blocks were synthesized (IDT) and assembled into pcDNA3 vector using NEBuilder to generate donor plasmids. Positive clones were screened by PCR and confirmed by Sanger sequencing. Presence of epitope tags was validated by western blot. ATRX KO mESCs were generated by co-transfecting CRISPR/Cas9 plasmids with gRNAs targeting the 5′ and 3′ ends of ATRX (Supplementary Table S1). ATRX KO clones were screened by western blot. Stable ATRX knockdown (ATRX KD) cell lines were generated as described previously (8) and validated by western blot.

### Western blot and cell fractionation

Nuclear fractionation was performed as described previously (23,24). Quantification of the total signal for protein distribution across cytosol, nuclear and chromatin-bound fractions was computed using ImageJ. Each fraction is reported relative to sum intensity across all fractions. Lists of antibodies used in this study can be found in Supplementary Table S1.

### Immunofluorescence

Immunostaining of mESCs was performed as described previously (29). For immunostaining of β3-tubulin in NPCs, mESCs were seeded on the coverslips for differentiation. On the day 6 of NPC differentiation, cells were fixed and processed for immunostaining (29).

### CUT&RUN, ChIP and RNA-Seq library generation

CUT&RUN was performed as previously described (30,31) using EZH2, H3K27me3 and IgG antibodies on 5 million cells per sample. DNA obtained from Drosophila melanogaster S2 cells was sonicated to 200bp and 1ng was added to each CUT&RUN sample as a heterologous spike-in control. ChIP was performed as previously described (32) using 9 μg of ATRX antibody (21) and 6 million cells per sample. For RNA-sequencing, RNA samples were extracted from Day 0 mESCs and Day 6 NPCs using Trizol reagent (Invitrogen) and subjected to DNase digestion with Turbo DNase (Ambion AM2238), then rRNA-depleted using FastSelect -rRNA HMR (Qiagen) and converted to cDNA using Ultra II Directional RNA Library Prep Kit (NEB E7760). DNA and cDNA samples were end-repaired using End-Repair Mix (Enzymatics), A-tailed using Klenow exonuclease minus (Enzymatics), purified with MinElute columns (Qiagen), and ligated to Illumina adapters (NEB #E7600) with T4 DNA ligase (Enzymatics). Size selection for fragments >150 bp was performed with AMpure XP (Beckman Coulter). Libraries were PCR amplified with barcoded adapters for Illumina sequencing (NEB #E7600) using Q5 DNA polymerase (NEB #M0491) and purified with MinElute. Sequencing was performed on a NextSeq 500 instrument (Illumina) with 38 × 2 paired-end cycles.

### Sequencing alignment and processing

CUT&RUN and ChIP-seq reads were mapped to the mm10 mouse genome with Bowtie2 version 2.2.9 using default parameters and paired-end setting (33). Peaks were called with MACS2 2.2.184 using the parameters ‘-f BAMPE -g mm –keep-dup all’ for EZH2 and H3K27me3 CUT&RUN and ‘-f BAMPE -g mm –keep-dup 1 –broad’ for ATRX ChIP-Seq (34). For CUT&RUN, peaks present in two replicates and that were not in an unknown contig or blacklisted region were kept as the consensus peakset. For ChIP-Seq, peaks in either replicate and that were not in an unknown contig or blacklist region were combined. BigWig tracks were generated using the bamCoverage function in deepTools version 3.4.1 using the parameters ‘–binSize 5 –extendReads –blackListFileName’, which removes a known set of ENCODE blacklist regions (35). For CUT&RUN spike-normalized BigWig tracks, the ‘–scaleFactor’ option was used to introduce a normalization factor, calculated based on the number of reads aligning to the Drosophila genome. For ATRX ChIP-Seq RPM BigWig tracks, the ‘—ignoreDuplicates’ option was used to remove PCR duplicates. Signal plots of normalized tracks were generated using the computeMatrix and plotProfile functions in deepTools (36). Heatmaps were generated using the plotHeatmap function in deepTools. Read density values used for scatterplots and boxplots were calculated using the multiBigwigSummary function in deepTools. Differential binding analysis of CUT&RUN data was performed in R version 3.6.1 using DiffBind version 2.12.0 (http://bioconductor.org/packages/release/bioc/html/DiffBind.html). Sites of differential occupancy was called using the edgeR method and an FDR cutoff of 0.05. Genomic annotation of peaks and association of gene symbols was performed using ChIPseeker version 1.20.0 (37).

RNA-Seq data were aligned using STAR version 2.7.3 (38). RSEM version 1.3.3 was used to obtain estimated counts (39). Differential analysis of RNA-Seq data was performed in R version 4.1.1 using the packages limma version 3.50.1 and edgeR version 3.36.0. For RNA-Seq differential analysis, genes were filtered using the edgeR built-in function ‘filterByExpr’. A differentially expressed gene was defined using the cutoffs of adjusted *P*-value ≤0.05 and absolute log_2_ fold change >1. Annotation of ATRX and EZH2 peaks and association of gene symbols was performed using ChIPseeker version 1.30.3 (37). A gene was defined as an ATRX target if an ATRX ChIP-Seq peak was within 3kb upstream or downstream of the gene body, and anEZH2 target if an EZH2 CUT&RUN peak was within 3 kb upstream or downstream of the transcription start site. RNA-Seq *Z*-scores were calculated using TPM values across all replicates and using sample groups as indicated in figure legends. RNA-Seq heatmaps were generated using the pheatmap R package. Venn diagrams of gene list overlaps were generated using Ensembl IDs. Enrichment analysis of gene sets was performed with DAVID, a browser-based application, using Ensembl IDs as input (40,41).

## RESULTS

### Characterization of ATRX PHD finger and helicase domain mutant mESCs

**Figure 1.**
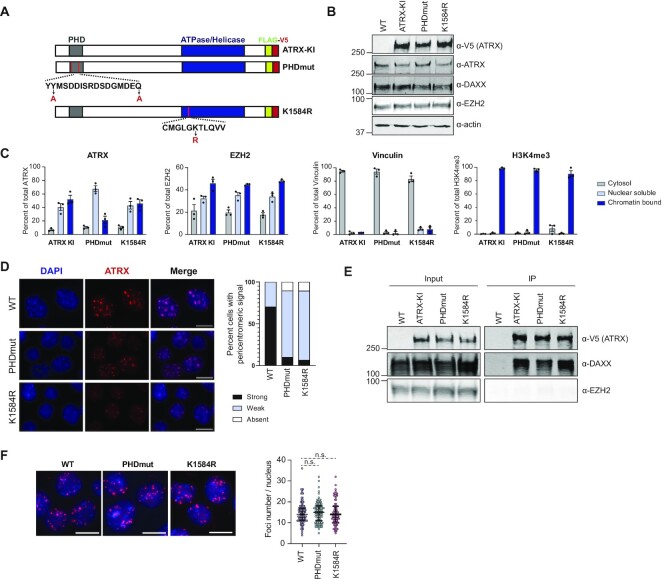
Characterization of ATRX PHD finger and helicase domain mutant mESCs. (**A**) Schematic of Flag and V5 tagged ATRX-KI, PHDmut, and K1584R proteins. PHD fingers are shown in grey and the helicase domain in blue. Conserved amino acid residues within each region are shown with the specific mutations in red. (**B**) Western blot for ATRX, DAXX and EZH2 in nuclear extract from WT, ATRX-KI, PHDmut and K1584R mESCs. V5 antibody was used to detect tagged proteins in ATRX-KI, PHDmut and K1584R mESCs. Actin serves as a loading control. (**C**) Quantification of ATRXKI, PHDmut, and K1584R distribution between cytosolic, nuclear soluble, and chromatin-bound fractions in mESC. Band intensity was measured with ImageJ for three independent Western blots. Vinculin was used as cytosolic marker. EZH2 and H3K4me3 served as nuclear soluble and chromatin bound fraction markers. Bar charts present mean values ±SEM. (**D**) Left: immunostaining of WT, PHDmut, and K1584R mESCs for ATRX (red) and DAPI (blue). Scale bar = 10 μm. Right: quantification of nuclei (*n* = 200–300) that show strong (black), weak (blue) or absent (white) ATRX signal at pericentromeres. (**E**) Flag immunoprecipitation of ATRX in WT, ATRX-KI, PHDmut and K1584R mESCs. Inputs and immunoprecipitates were analyzed for ATRX, DAXX and EZH2 with antibodies as labeled on the right. (**F**) Left: representative image of proximity-ligation assay (PLA), showing in situ co-localization between ATRX and DAXX (red) in WT, PHDmut and K1584R mESC. DNA was stained with DAPI (blue). Scale bar = 10 μm. Right: quantification of PLA foci number per nucleus (*n* = 100). Lines on scatter plots represent 25th-, median and 75th-percentile. Statistical significance was determined by Welsh's test.

ATRX is an X-linked gene and therefore only a single copy is present in diploid male cells. CRISPR/Cas9 targeted mutation thus enables us to exclusively express mutant ATRX proteins in male ESCs. The ATRX PHD finger and helicase domain are highly conserved across species (Supplementary Figure S1A and B). The crystal structure of the ATRX PHD domain revealed that this region forms two binding pockets, one that recognizes H3K9me2 or H3K9me3 and a second that binds H3K4me0 (17–19). Based on published structural data that described the molecular basis of ATRX-PHD finger and histone interactions, we mutated two critical residues, tyrosine 202 and glutamine 217, to alanine (Y202A and Q217A) (Supplementary Figure S1A). The Y202 residue forms hydrogen bonds that stabilize the H3K9me3 binding pocket, while Q217 is important for recognition of unmodified H3K4. We expected that mutation of these two conserved residues would significantly destabilize or even abolish ATRX interactions with histone H3. We separately generated an mESC line that contained the lysine 1584 to arginine substitution (K1584R) that was shown to abolish ATP hydrolysis activity (Supplementary Figure S1B) (21). We confirmed the presence of desired point mutations within the PHD finger and helicase domains by Sanger sequencing (Supplementary Figs. 1C and 1D). We simultaneously introduced FLAG and V5 epitope tags at the C terminus to facilitate immunoprecipitation and detection. As a control, we generated WT ATRX FLAG-V5 knock-in (KI) mESCs. We refer to these cell lines as ATRX-KI, PHDmut and K1584R (Figure 1A).

Previous studies using ATRX syndrome patient cell lines show that while some ATRX missense mutations result in reduced protein levels, others do not significantly impact protein stability (21). To determine whether PHDmut and K1584R mESCs express similar levels of ATRX as parental mESCs (WT), we examined ATRX protein levels in nuclear extracts from all three cell lines (Figure 1B). We also examined the protein levels of DAXX, with which ATRX forms a complex for histone H3.3 deposition. Anti-V5 western blot confirmed the presence of a V5 epitope tag in ATRX-KI, PHDmut, and K1584R, but not in the parental cell line. Both ATRX and DAXX proteins were expressed at similar levels across all three cell lines. Next, we tested whether mutations in the PHD finger and the helicase domain affect its cellular localization. ATRX normally localizes to the nucleus and shows strong association to chromatin. We confirmed that expression level of ATRX in ATRX-KI, PHDmut, and K1584R mESCs was comparable (Supplementary Figure S1E, left) and fractionated cells into cytosolic, nuclear soluble, and chromatin bound fractions, then examined the distribution of ATRX. Under our experimental conditions, ATRX is nuclear in all cell lines and is equally distributed between nuclear soluble and chromatin bound fractions in ATRX-KI. Interestingly, PHDmut shows reduced levels in the chromatin bound fraction and accumulation in the nuclear soluble fraction (Figure 1C, Supplementary Figure S1E, right). This is consistent with PHD finger mutations abolishing histone interactions that would result in reduced chromatin localization. K1584R mutation does not appear to affect ATRX cellular localization, and it behaves similar to ATRX-KI. As a control, EZH2, a component of the polycomb repressive complex 2 (PRC2), does not show any change in localization in either mutant, and remains predominantly nuclear as expected (Figure 1C, Supplementary Figure S1E, right). Vinculin, a cytoplasmic protein, was present in the cytosolic fraction, and histone H3 trimethylated at lysine 4, a chromatin associated mark for active transcription, remained in the chromatin bound fraction in all cell lines (Figure 1C, Supplementary Figure S1E, right).

We next examined the spatial distribution of ATRX within the nucleus by immunofluorescence microscopy. ATRX normally localizes to pericentromeres, which are visible as DAPI-dense regions in the nuclei of mESCs. In WT mESCs, ATRX signal is clearly enriched at pericentromeres in over 70% of nuclei examined (Figure 1D). In contrast, despite being expressed at similar levels as WT (Figure 1B), both ATRX PHDmut and K1584R show reduced localization to pericentromeres. Mutant protein redistribution does not result in overall increased nuclear signal. Interestingly, K1584R remains chromatin-bound (Figure 1C, Supplementary Figure S1E) despite its observed loss at pericentromeres, suggested it may be spatially redistributed on chromatin away from pericentromeric regions. We also examined whether ATRX mutations influence the heterochromatin associated H3K9me3 histone modification at pericentromeres. H3K9me3 enrichment was not significantly affected in ATRX knock out (KO) (7) or K1584R mESCs and only minimally changed in PHDmut (Supplementary Figure S1F). To exclude the possibility that mutant protein mislocalization is because of protein misfolding, we examined the interaction between ATRX and the histone chaperone DAXX. Flag immunoprecipitation in PHDmut and K1584R mESCs confirmed that ATRX PHD finger and helicase domain missense mutations do not compromise interactions with DAXX (Figure 1E). Consistent with previous reports (42), we could not identify interactions between ATRX and EZH2 in nuclear extracts, suggesting context specific interactions in vivo (Figure 1E). To quantify ATRX and DAXX interactions in situ, we performed proximity ligation assays (PLA) in WT, PHDmut and K1584R mESCs. We identified ATRX-DAXX interactions in all three cell lines (Figure 1F, left). Quantification of PLA foci showed that ATRX-DAXX interactions were not significantly different between WT and PHDmut and K1584R mESCs (Figure 1F, right). Altogether, our results show that disease causing missense mutations in the PHD finger or helicase domains of ATRX differentially impact protein localization.

### ATRX mutations result in distinct neurodifferentiation defects

**Figure 2.**
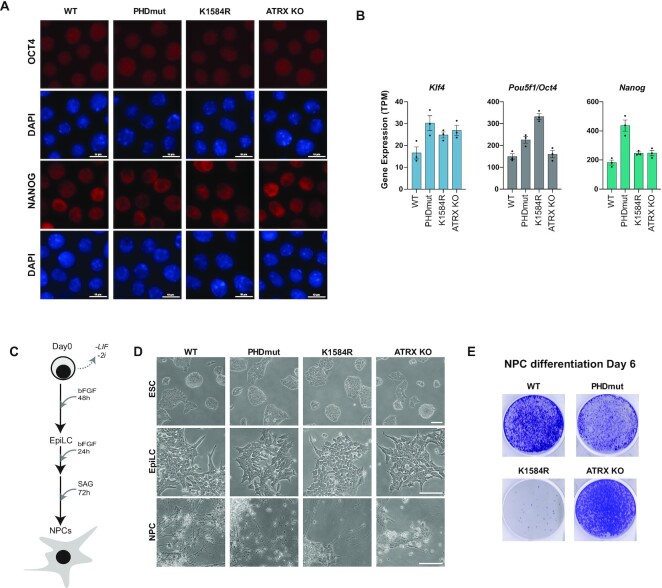
ATRX PHD finger and helicase domain mutants display neurodifferentiation defects. (**A**) Immunostaining of WT, PHDmut, K1584R and ATRXKO for OCT4 and NANOG (red) and DAPI (blue) in mESC. Scale bar = 10 μm. (**B**) Bar chart showing expression of *Klf4*, *Pou5f1 (Oct4)*, and *Nanog* as transcripts per million (TPM) in the mESC state. Circles indicate individual biological replicates. Data are presented as mean values ± SEM. (**C**) Schematic of neural differentiation. Withdrawal of LIF/2i and addition of basic Fibroblast Growth Factor (bFGF) results in the generation of EpiLCs. Addition of Smoothened Agonist (SAG) to EpiLCs promotes NPC formation. (**D**) Representative phase contrast images of WT, PHDmut, K1584R and ATRX KO at the mESC, EpiLC and NPC stages. Scale bar = 100 μm. (**E**) Representative images of crystal violet staining assay for cell populations in WT, PHDmut, K1584R and ATRX KO at day 6 of NPC differentiation.

ATRX mutations in the PHD finger and the helicase domains cause ATRX syndrome (25). To test if ATRX mutations affect neuronal differentiation in a mouse embryonic stem cell model, we characterized the effect of mutations in undifferentiated cells and upon differentiation into neural progenitors. We tested the expression of pluripotency markers OCT4 and NANOG and found that both proteins showed similar nuclear staining in all cell lines (Figure 2A). ATRX PHDmut, K1564R, and ATRX knock out mESCs (Supplementary Figure S2A) appear indistinguishable from WT mESCs (Figure 2D). Similarly, RNA-seq analysis in WT, PHDmut, K1584R, and ATRX KO mESCs showed that in the stem cell state, WT, PHDmut, K1584R and ATRX KO express high levels of pluripotency markers such as *Klf4*, *Pou5f1* and *Nanog* (Figure 2B).

Next, we induced differentiation of WT, ATRX mutants, and ATRX KO mESCs into neural progenitor cells (NPCs). We adopted a two-step directed-differentiation protocol that guides cells toward the ectoderm lineage through an epiblast-like cell (EpiLC) intermediate, and ultimately results in the generation of NPCs (Figure 2C) (43). Wildtype, PHDmut, K1584R and ATRX KO mESCs appear identical and form compact colonies in the undifferentiated state (Figure 2D, top row). We initiated neural differentiation by withdrawal of leukocyte inhibitory factor (LIF) and glycogen synthase and MEK1/2 inhibitors, and addition of basic fibroblast grown factor (bFGF) (Figure 2C). After 48 hours at the EpiLC stage, colonies flatten and cell boundaries become more apparent in all cell lines (Figure 2D, middle row). Further 24 h incubation with bFGF and subsequent addition of smoothened agonist (SAG) at the 72-h timepoint induces further differentiation into NPCs. At day 6 of neurodifferentiation, WT NPCs flatten and produce long extensions resembling neurites (Figure 2D, bottom row). In comparison, NPCs derived from ATRX KO mESCs remain small and produce few, if any, outgrowths, suggesting that NPC differentiation is impaired when ATRX is depleted. Both PHDmut and K1584R mutants also show reduced and delayed differentiation compared to WT. Notably, the K1584R mutant shows significantly increased cell death compared to WT and PHDmut (Figure 2D, bottom row). To visualize differences in cell numbers during differentiation, we stained cells with crystal violet at the EpiLC and NPC stages. Equal number of mESCs were seeded on plates prior to initiation of neural differentiation. At the EpiLC stage, PHDmut and ATRX KO containing wells appear to have similar cell numbers as WT and K1584R shows slightly reduced cell numbers (Supplementary Figure S2B). Interestingly, K1584R continues to show significant cell death through differentiation into NPCs and very few colonies survive at the NPC stage (Figure 2E) and surviving colonies show fewer neurite outgrowths compared to WT (Figure 2D).

### ATRX PHDmut and K1584R mutations deregulate unique gene groups in mESCs and NPCs

**Figure 3.**
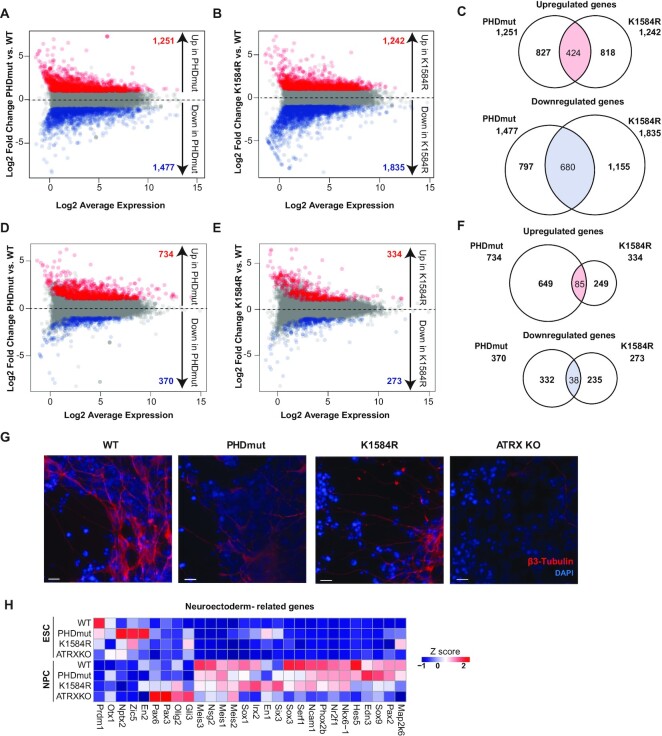
ATRX PHDmut and K1584R mutations deregulate unique gene groups in mESCs and NPCs. (**A**) MA plot of RNA-seq expression of 13,958 genes between WT and PHDmut mESCs. For all MA plots in this figure, red dots and numbers indicate differentially expressed genes that are upregulated in mutant cells (adjusted *P*-value ≤0.05, log_2_ fold change > 1); blue dots and numbers indicate differentially expressed genes that are downregulated in mutant cells (adjusted *P*-value ≤ 0.05, log_2_ fold change < –1). (**B**) MA plot of RNA-seq expression of 13,347 genes between WT and K1584R mESCs. (**C**) Venn diagram showing overlaps between upregulated and downregulated differentially expressed genes in PHDmut mESCs and K1584R mESCs (red and blue dots in Figure 3A and B). Total numbers of differentially expressed genes and overlaps are shown. (**D**) MA plot of RNA-seq expression of 14,879 genes between WT and PHDmut NPCs. (**E**) MA plot of RNA-seq expression of 15,079 genes between WT and K1584R NPCs. (**F**) Venn diagram showing overlaps between upregulated and downregulated differentially expressed genes in PHDmut NPCs and K1584R NPCs (red and blue dots in D and E). Total numbers of differentially expressed genes and overlaps are shown. (**G**) Immunostaining of WT, PHDmut, K1584R and ATRXKO for β3-Tubulin (red) and DAPI (blue) at day6 of NPC differentiation. Scale bar = 10 μm. (**H**) Heat map of expression select neuroectoderm related genes in ESC and NPC. Each column represents the mean *Z*-score for three independent biological replicates from RNA-seq.

We performed RNA-sequencing in WT, PHDmut, K1584R and ATRX KO mESCs to identify shared and unique gene expression signatures associated with specific ATRX mutations. Principal component analyses indicate that overall gene expression profiles are similar across three replicates of the same cell line (Supplementary Figure S3A). Interestingly, PHDmut and ATRX KO RNA-seq samples show higher concordance in gene expression signatures as evidenced by their clustering closer to each other and away from WT and K1584R. Differential gene expression analysis revealed that 1,251 genes were upregulated and 1,477 were downregulated in PHDmut compared to WT (Figure 3A, Supplementary Table 2). Similarly, 1242 genes were upregulated and 1835 were downregulated in K1584R compared to WT (Figure 3B, Supplementary Table 2). In ATRX KO mESCs, 1456 were upregulated and 1825 were downregulated compared to WT (Supplementary Figure S3B, Supplementary Table 2). Overlap of differentially expressed genes (DEGs) between all three cell types identified both shared and unique gene expression patterns between the mutants (Supplementary Figure S3C). To identify specific differences between PHDmut and K1584R, we compared DEGs and found that 424 (∼30%) up- and 680 (∼45%) down-regulated genes overlap in PHDmut and K1584R (Figure 3C). Most of the shared differentially expressed genes across PHDmut, K1584R, and ATRX KO (Supplementary Figure S3C) had general roles in chromatin organization, which can have important effects on developmental gene expression (Supplementary Figure S3D).

We also performed RNA-seq in WT, PHDmut, K1584R and ATRX KO at the NPC stage. Principal component analyses indicate that overall gene expression profiles are similar across three replicates of the same cell line (Supplementary Figure S4A). However, at the NPC stage, overall gene expression signatures in ATRX KO samples are distinct from WT, PHDmut and K1584R as seen by their separation on the PCA plot (Supplementary Figure S4A). For a comprehensive view of gene expression changes in ATRX PHDmut and K1584R during neuronal differentiation, we identified DEGs in PHDmut and K1584R NPCs compared to WT NPCs (Supplementary Table S2). Our analysis uncovered 1,104 genes that are differentially expressed in PHDmut compared to WT, 607 genes that are differentially expressed in K1584R compared to WT (Figure 3D, E), and 3,989 genes that are differentially expressed in ATRX KO compared to WT (Supplementary Figure S4C). Overlap of DEGs in NPCs between all three cell types showed that ATRX KO cells deregulate many more unique genes compared to PHDmut or K1584R, consistent with PCA results (Supplementary Figure S4B). Down-regulated genes in ATRX KO NPCs had specific roles in biological processes related to nervous system development and neuron differentiation (Supplementary Figure S4D). To determine if PHDmut and K1584R mutations deregulate unique genes in NPCs, we compared the overlap between genes upregulated in PHDmut to those upregulated in K1584R. Only 85 genes are upregulated in both PHDmut and K1584R NPCs, with the remaining majority uniquely upregulated in PHDmut (649) or K1584R (249) NPCs (Figure 3F). Similarly, downregulated genes also do not show a large overlap (Figure 3F). Our results indicate that ATRX PHDmut and K1584R mutations affect the expression of distinct genes at the NPC stage and thus may contribute to defective neurodifferentiation through different pathways. To identify the specific biological processes deregulated in each mutant, we performed gene ontology analyses of uniquely up- and downregulated genes in PHDmut and K1584R (Supplementary Figure S4E, F). Genes that are upregulated only in K1584R, or downregulated only in PHDmut, have functions directly related to synapse organization, axon guidance, and nervous system development. Genes that are upregulated exclusively in PHDmut NPCs have roles in circulatory system development and finally, downregulated genes in K1584R regulate glucose metabolic processes that have critical function in neuronal and non-neuronal cell maintenance.

Next, we examined expression levels of pluripotency and neuronal markers in WT, PHDmut, K1584R and ATRX KO mESCs and NPCs. Upon differentiation, pluripotency markers are downregulated similarly in WT, PHDmut, K1584R and ATRX KO, indicating that ATRX mutations or loss do not affect exit from pluripotency or entry into differentiation (Supplementary Figure S5A). We also examined expression levels of *Nestin*, *Actl6b* and *Tubb3* neuronal markers. As expected, neuronal markers are expressed at very low levels in the ESC stage in WT and all mutants. At the NPC stage, these markers are upregulated as expected in WT cells. However, expression of these genes is markedly reduced in PHDmut, K1584R and ATRX KO NPCs compared to WT (Figure 3G, Supplementary Figure S5B), consistent with delayed and reduced neurodifferentiation. This reduced induction was also observed in a broader panel of genes with specific roles in neuroectoderm development (Figure 3H). Interestingly, even within the neuroectoderm gene cluster PHDmut and K1584R show distinct effects on induction at the NPC stage. ATRX KO shows the most drastic effects with most genes not being appropriately induced in NPCs (Figure 3H), consistent with more aberrant gene expression in ATRX KO in NPCs compared to PHDmut and K1584R (Supplementary Figure 4A and B). We also examined if ATRX mutations derepress endoderm and mesoderm genes and found that distinct subsets of mesoderm and endoderm markers are upregulated in PHDmut and ATRX KO at the NPC stage (Supplementary Figure S5C, D). However, we did not observe activation of all meso- and endoderm genes that would promote efficient differentiation along these lineages. These results suggest that ATRX PHD finger mutations and ATRX knock out may compromise silencing of some endodermal genes that then become spuriously activated upon differentiation. Overall, our results indicate that PHD finger and helicase domain mutations in ATRX contribute to neurodifferentiation defects through the dysregulation of distinct gene expression pathways that ultimately manifest as unique developmental outcomes.

### ATRX PHDmut and K1584R show opposing effects on ATRX localization to targets

**Figure 4.**
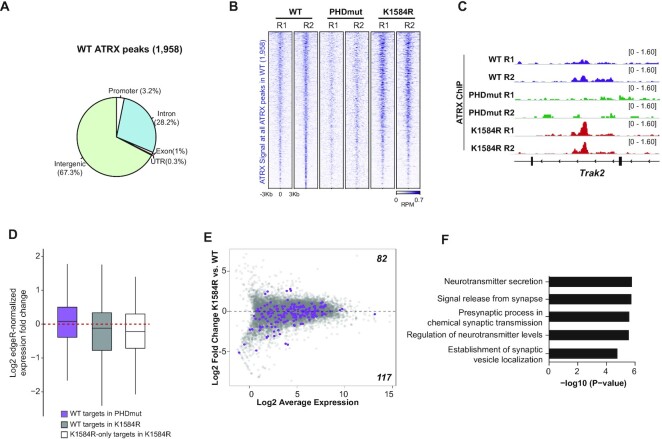
PHDmut and K1584R mutations have distinct effects on genome wide ATRX binding. (**A**) Genomic distribution of 1,958 ATRX ChIP-Seq peaks called across two replicates in WT mESCs. (**B**) Heatmap of ATRX ChIP-Seq signal across 1,958 ATRX peaks in WT, PHDmut and K1584R mESCs. Reads per million, RPM. (**C**) Genome browser view of the *Trak2* gene showing ATRX ChIP signal (reads per million, RPM) in WT, PHDmut and K1584R mESCs. (**D**) Boxplots showing log_2_ edgeR-normalized expression fold change of WT ATRX target genes in PHDmut compared to WT (purple); WT ATRX target genes in K1584R compared to WT (gray); and K1584R-only ATRX target genes in K1584R compared to WT (white). Box, 25th percentile—median—75th percentile. Whiskers extend to 1.5× interquartile range; outliers not displayed. (**E**) MA plot of RNA-Seq expression for 13,347 genes between WT and K1584R mESCs. Purple dots indicate K1584R-only ATRX target genes that overlap an ATRX peak identified only in K1584R mESCs. (**F**) Top 5 most significantly enriched processes in K1584R-only ATRX targets.

To gain a mechanistic understanding of how ATRX mutations affect neurodifferentiation, we first tested how ATRX mutant proteins localize across the genome. We performed ATRX ChIP-seq in WT, PHDmut, and K1584R mESCs using antibodies that we have generated and previously characterized (23). We found high positive correlation across replicates (Supplementary Figure S6A) and identified 1,958 ATRX peaks from two ATRX ChIP-seq replicates from WT mESCs (Figure 4A). We found that ∼33% of ATRX peaks localized to genes and ∼67% to intergenic sites. Comparison of ATRX binding in WT, PHDmut, and K1584R showed that ATRX PHDmut enrichment was decreased at ATRX targets, while in contrast, ATRX K1584R showed higher enrichment at the same sites (Figure 4B, C). This observation is in line with previous biochemical data showing that ATRX binding to DNA and RNA is altered by ATP hydrolysis and that the inability to hydrolyze ATP increased ATRX binding to nucleic acids (8).

The observed changes in ATRX occupancy in K1584R and PHDmut prompted us to examine their consequence to gene expression. We defined ‘ATRX target genes’ as those containing an ATRX peak in the promoter or gene body in WT mESCs. While ATRX has function in facilitating transcriptional elongation at some guanine-rich genes (44), it has been more well-studied in the context of transcriptional repression (2,7,45). Therefore, we expected many ATRX target genes to become derepressed upon loss of ATRX enrichment in PHDmut mESCs. Indeed, 60% of ATRX target genes showed slightly increased expression in PHDmut compared to WT (Figure 4D). Next, we examined WT ATRX targets in K1584R mESCs, which show accumulation of mutant protein. ATRX genes showed decreased expression compared to WT (55%, Figure 4D), again consistent with ATRX’s role in repression. We also found that K1584R accumulated at sites that were not identified as ATRX targets in WT mESCs. These *de novo* K1584R-only ATRX targets show decreased expression in K1584R compared to WT (Figure 4D and E) and are enriched for processes important for nervous system function (Figure 4F).

### ATRX PHDmut and K1584R mutations affect PRC2 localization and polycomb gene expression

**Figure 5.**
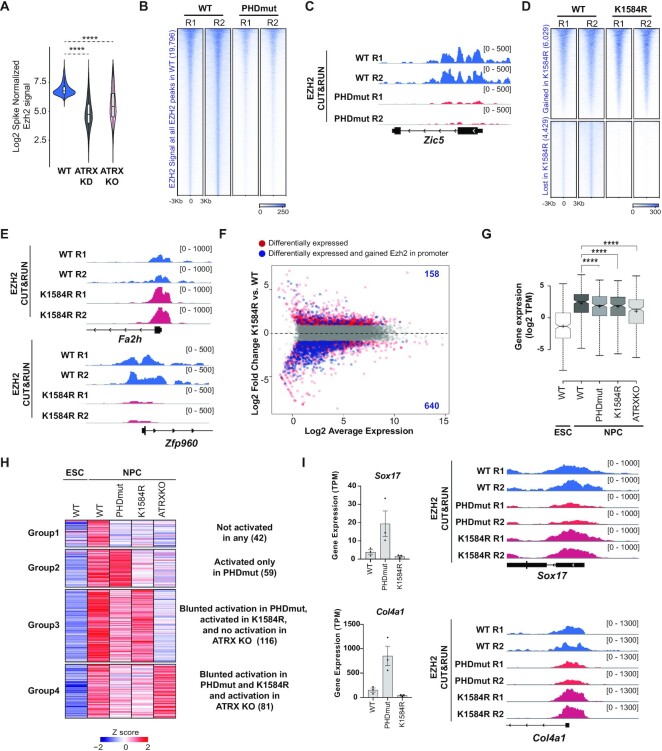
ATRX PHDmut and K1584R mutations differentially alter PRC2 occupancy. (**A**) Violin plot of spike-normalized EZH2 CUT&RUN signal in WT, ATRX KD and ATRX KO mESCs. Box, 25th percentile—median—75th percentile. Whiskers extend to 1.5× interquartile range; outliers not displayed. (**B**) Heatmap of spike-in normalized EZH2 CUT&RUN signal across 19 796 WT EZH2 peaks in WT and PHDmut mESCs. (**C**) Genome browser view of the *Zic5* gene showing spike-in normalized EZH2 CUT&RUN signal in WT and PHDmut mESCs. (**D**) Heatmap of spike-in normalized EZH2 CUT&RUN signal in WT and K1584R mESCs across EZH2 peaks that are gained (6,029) or lost (4,429) in K1584R compared to WT. (**E**) Genome browser view of the *Fa2h* and *Zfp960* genes showing spike-in normalized EZH2 CUT&RUN signal in WT and K1584R mESCs. (**F**) MA plot of RNA-seq expression of 13,347 genes between WT and K1584R mESCs. Red dots indicate differentially expressed genes (adjusted *P*-value ≤ 0.05, absolute log_2_ fold change > 1); blue dots and numbers indicate differentially expressed genes with gained promoter EZH2 in K1584R. (**G**) Boxplots showing expression of poised enhancer related genes that are activated in NPCs. Box represents 25th, median and 75th percentile. The whiskers correspond to minimum and maximum values. The Wilcoxon signed-rank test was used for calculation significance calculation, ****P < 0.001. (**H**) Heatmap of expression of 298 poised enhancer related genes that are induced upon differentiation and grouped by *k*-means clustering of *Z*-scores at the NPC stage in WT, PHDmut, K1584R and ATRX KO. Each column represents the mean *Z*-score for three independent biological replicates from RNA-seq. *Z*-scores were computed across WT, PHDmut, K1584R and ATRX KO mESCs and NPCs. (**I**) Left: bar chart showing expression of *Sox17* and *Col4a1* as transcripts per million (TPM) in the NPC state. Circles indicate individual biological replicates. Data are presented as mean values ± SEM. Right: genome browser view of the *Sox17* and *Col4a1* genes showing spike-in normalized EZH2 CUT&RUN signal in WT, PHDmut and K1584R mESCs.

ATRX regulates PRC2 localization genome-wide (8,23). ATRX knock down (KD) in mouse embryonic fibroblasts (MEF) decreases PRC2 enrichment at its targets. To test if ATRX regulates PRC2 localization in mESCs, we performed CUT&RUN to examine the genomic occupancy of EZH2, the catalytic subunit of the PRC2 complex, in wildtype, ATRX KD and KO mESCs. We called EZH2 peaks across two WT replicates and found that many EZH2 peaks show centralized enrichment of ATRX (Supplementary Figure S6B). We also found that loss of ATRX results in decreased EZH2 at these peaks (Figure 5A, Supplementary 6C). Next, we performed EZH2 CUT&RUN to test the effects of ATRX PHDmut and K1584R on PRC2. Similar to ATRX KD and KO, EZH2 enrichment is decreased across all EZH2 peaks in ATRX PHDmut mESCs (Figure 5B, C). Interestingly, EZH2 CUT&RUN in K1584R showed that while some sites show clear decrease in EZH2 (Supplementary Figure S6D, bottom of heatmap), others show no change or even a slight increase. To distinguish between sites that gain or lose PRC2 in ATRX K1584 mutant mESCs, we identified differentially bound EZH2 regions in K1584R compared to WT. We found that in K1584R, 6029 sites gain and 4429 sites lose EZH2 (Figure 5D and E). Most EZH2 gains occurred at promoter regions (79%), while sites that lose EZH2 were equally spread between promoter, genic and intergenic regions (Supplementary Figure S6E). Gene expression analyses revealed that PRC2 targets with gained promoter EZH2 in K1584R show decreased expression (Figure 5F). However, the loss of PRC2 from promoters did not necessarily result in derepression of target genes (Supplementary Figure S6F), in line with previous studies showing that most polycomb targets are not upregulated in response to PRC2 loss or inhibition and may require the action of additional transcriptional activators (46–48). This is in stark contrast to the larger number of polycomb targets that are derepressed in PHDmut (Supplementary Figure S6G). It is possible that loss of ATRX from chromatin in PHDmut impacts additional pathways that result in activation of polycomb targets. Genes showing increased EZH2 in K1584R and reduced expression were enriched in processes related to neuronal function and development (Supplementary Figure S6H).

In addition to genes, PRC2 was previously shown to regulate a subset of enhancers termed ‘poised enhancers’. Poised enhancers regulate the expression of a subset of genes with critical roles in neurodevelopment by activating them upon differentiation (49). Poised enhancers are enriched for components of the PRC2 complex such as EZH2 and SUZ12, and its catalytic mark H3K27me3 and depleted for marks of active transcription such as H3K27ac (Supplementary Figure S7A). PRC2 is suggested to maintain topological associations between poised enhancers and their targets in mESCs (49). Consequently, PRC2 depletion results in a blunted activation of target genes upon differentiation due to loss of topological association in the undifferentiated state. Using RNA-seq data from WT mESCs and NPCs, we identified 298 poised enhancer related genes that become activated in the NPC stage (Supplementary Figure S7B, Supplementary Table S3). Using independently published datasets, we confirmed that these activated genes show reduced expression in NPCs upon acute depletion of SUZ12, a core PRC2 complex component (Supplementary Figure S7C) (48). Next, we analyzed the expression of these 298 poised enhancer related genes in ATRX mutant and KO NPCs. ATRX KO NPCs show blunted expression of these genes, further strengthening a functional connection between ATRX and the PRC2 complex (Figure 5G, Supplementary Figure S7D). Similar to ATRX KO, both PHDmut, and K1584R NPCs also show blunted induction of poised enhancer regulated genes (Figure 5G, Supplementary Figure S7D). Interestingly, further clustering of poised enhancer genes based on expression in NPCs identified distinct genes that are affected in PHDmut, K1584R, and ATRX KO mutants (Figure 5H, Supplementary Table S3). Poised enhancer related genes were similarly deregulated in Suz12-AID and ATRX KO mESCs (Supplementary Figure S7E). Genes in all clusters were enriched in various functions critical for nervous system regulation (Supplementary Figure S7F).

Finally, we examined if mislocalization of PRC2 can contribute to derepression of some lineage specification genes. We examined EZH2 enrichment at *Sox17* and *Col4a1*, two endodermal lineage genes that show increased expression in the NPC stage in PHDmut, but not in K1584R (Figure 5I and Supplementary Figure S5D). We found that EZH2 is decreased at *Sox17* and *Col4a1* promoters in PHDmut mESCs and slightly increased at these sites in K1584R mESCs, linking specific ATRX mutations to PRC2 deregulation and aberrant developmental gene expression. Together, our results indicate that ATRX mutations in either the PHD or helicase domains contribute to neuronal differentiation defects by affecting the expression of distinct poised enhancer-regulated genes, which have unique roles in early brain development.

## DISCUSSION

In this study, we engineered mESCs to exclusively express mutant ATRX proteins containing missense mutations in the PHD finger or helicase domains, which compromise its histone binding or ATP hydrolysis activities respectively, to examine how specific ATRX mutations contribute to neurodifferentiation defects. We find that while ATRX mutant proteins are expressed at similar levels as WT protein in mESCs and interact with the DAXX histone chaperone, their localization to chromatin is differentially impacted (Figure 1). ATRX PHDmut shows reduced localization to chromatin while K1584R behaves more similarly to WT ATRX. We found that both PHDmut and K1584R mESCs show delayed and reduced differentiation into NPCs (Figure 2). RNA-seq analyses identified distinct changes in gene expression in PHDmut, K1584R, and ATRX KO at the ESC and NPC stages and all ATRX mutants show decreased expression of neural lineage markers at the NPC stage (Figure 3). Interestingly, PHDmut and K1584R mutations show distinct effects on ATRX localization, with PHD mutations reducing ATRX enrichment at targets and K1584R accumulating at ATRX targets (Figure 4). This behavior may point to dominant negative effects in helicase domain mutations that compromise the ATPase activity of ATRX. The distinct effects of PHDmut and K1584R are also observed with respect to PRC2 localization (Figure 5). PRC2 is decreased at most polycomb targets in PHDmut and shows both increase and decrease at specific sites in K1584R. PRC2 increase is associated with gene downregulation, while its loss does not have a clear activation effect on target genes. PHDmut and K1584R also show blunted expression of poised enhancer related genes that play critical roles in neurodevelopment (Figure 5). Interestingly, comparison of effects of PHDmut, K1584R, ATRX KO, and Suz12 degradation shows that poised enhancer related genes were similarly deregulated in ATRX KO and Suz12 depleted mESCs.

Missense mutations in ATRX syndrome cluster in the PHD finger and helicase domains on the ATRX protein. However, analysis of ATRX protein levels from patient blood show that in many cases ATRX missense mutations result in reduced protein levels. Therefore, while haploinsufficiency may contribute to ATRX syndrome, the effects of the mutations themselves are obscured. Because ATRX protein levels are not affected by missense mutations in mESCs, our model provides a unique insight into ATRX syndrome and identifies specific pathways that are affected when histone binding or ATP hydrolysis functions are compromised. Our results suggest that gene expression signatures in PHD finger mutants are more similar to loss of ATRX, consistent with the reduced localization of PHDmut to chromatin. On the other hand, K1584R accumulates on chromatin at ATRX targets and at some new sites, implying a potential dominant negative mode of action. Interestingly, our results also reveal that ATRX through its chromatin localization suppresses differentiation toward endodermal lineages. This function appears to be independent of ATP hydrolysis and potentially chromatin remodeling activities as K1584R do not show aberrant activation of endoderm lineage genes. We speculate that ATRX interactions with silencing factors including HP1 and SETDB1 (7,19) may be affected in PHDmut contributing to developmental gene expression defects. In addition, ATRX is known to interact with the methyl CpG binding protein 2, MeCP2 (15) and recruits it to imprinted regions in the mouse forebrain (16). Therefore, it is also possible that loss of ATRX from specific regions in PHDmut can alter expression of genes through MeCP2 dependent mechanisms.

Our results have implications beyond neurodevelopmental disorders. In addition to ATRX syndrome, ATRX mutations occur frequently in neuroblastoma, glioblastoma, and osteosarcoma cancers (42,50–53). ATRX in frame deletion mutations that result in deletion of the PHD finger have been reported in neuroblastoma and osteosarcoma cancers (42,54,55). While most ATRX mutations in gliomas are loss of function due to truncations, missense mutations also occur with increased incidence in the helicase domain compared to other regions of the protein. Interestingly, our results suggest that mutations that abolish ATP hydrolysis activity of ATRX predominantly impact glycolysis, a metabolic pathway that drives growth and malignancy in solid tumors including neuro- and glioblastomas. The insights afforded by our studies in stem cells can be leveraged to develop new precision medicine approaches that target metabolic pathways to treat cancers with specific ATRX mutations.

## DATA AVAILABILITY

SUZ12 CUT&RUN data and SUZ12-AID related RNA-Seq data (48) were downloaded from GSE155997. WT mESC RNA-Seq data is available from our previous publication (27) at GSE160578. WT NPC RNA-Seq data is available from our previous publication (24) at GSE171401. Active, poised, and primed enhancer locations were obtained from (49). Sequencing data and processed tracks generated for this study have been deposited in the NCBI GEO as GSE193910.

## Supplementary Material

gkac683_Supplemental_Files

## Notes

Present address: Wenqing Ren, Tongji University Cancer Center, Shanghai 200092, China.
