# Supplementary material for: ATRX histone binding and helicase activities have distinct roles in neuronal differentiation
Source: Nucleic Acids Res. 2022 Aug 24;50(16):9162–74. doi: 10.1093/nar/gkac683 (PMC9458459; doi:10.1093/nar/gkac683)

Supplementary Figure Legends

Supplementary Figure 1. Generation of ATRX PHD finger and helicase domain mutant mESCs.

(A) Sequence conservation across species of residues within the PHD finger domain. Identical residues are marked by an asterisk (*). Conserved residues that are mutated in PHDmut are highlighted in red.

(B) Sequence conservation across species of residues within the helicase domain. Identical residues are marked by an asterisk (*). Conserved residue that is mutated in K1584R is highlighted in red.

(C) Comparison of mouse ATRX PHD finger cDNA sequence to Sanger sequencing results from the corresponding region in PHDmut. Mutated nucleotides are shown with a black arrowhead.

(D) Comparison of mouse ATRX helicase cDNA sequence to Sanger sequencing results from the corresponding region in K1584R. Mutated nucleotides are shown with a black arrowhead.

(E) Western blot in total nuclear extracts (left) and cytosolic, nuclear soluble, and chromatin-bound fractions (right) of ATRX-KI, PHDmut, and K1584R mESCs with antibodies as indicated.

(F) Left – Immunostaining of WT, PHDmut, K1584R, and ATRX KO mESCs for H3K9me3 (red) and DAPI (blue). Scale bar=10 µm. Right – Quantification of nuclei (n=200-300) for H3K9me3 signal at pericentromeres.

Supplementary Figure 2. WT, PHDmut, K1584R, and ATRX KO show similar growth at the EpiLC stage of differentiation.

(A) Western blot for ATRX and Actin in WT and 2 ATRX KO mESC clones.

(B) Representative images of crystal violet staining assay for cell populations in WT, PHDmut, K1584R, and ATRX KO in EpiLCs (day 2 of differentiation).

Supplementary Figure 3. ATRX PHDmut, K1584R, and ATRXKO alter unique gene groups in ESCs and NPCs.

(A) Principal component analysis of RNA-Seq gene expression (log2 TPM) in WT, PHDmut, K1584R, and ATRXKO mESCs.

(B) MA plot of RNA-seq expression of 14,225 genes between WT and ATRX KO mESCs.

(C) Venn diagram showing overlap between differentially expressed genes (adjusted p-value <= 0.05, log2 fold change > 1 or < -1) in PHDmut, K1584R and ATRXKO in mESCs.

(D) Top 5 significantly enriched processes (obtained from DAVID) in genes that are differentially expressed, uniquely or shared in PHDmut, K1584R, and ATRK KO mESC.

Supplementary Figure 4. ATRX PHDmut, K1584R, and ATRXKO alter unique gene groups in NPCs.

(A) Principal component analysis of RNA-Seq gene expression (log2 TPM) in WT, PHDmut, K1584R, and ATRXKO NPCs.

(B) Venn diagram showing overlap between differentially expressed genes (adjusted p-value <= 0.05, log2 fold change > 1 or < -1) in PHDmut, K1584R and ATRXKO in day 6 of NPC differentiation.

(C) MA plot of RNA-seq expression of 15,261 genes between WT and ATRX KO NPCs.

(D) Top 5 significantly enriched biological processes (obtained from DAVID) in genes that are upregulated (red) or downregulated (blue) in only ATRX KO NPCs.

(E) Top 5 significantly enriched processes (obtained from DAVID) in genes that are upregulated (red) or downregulated (blue) in only PHDmut NPCs.

(F) Top 5 significantly enriched processes (obtained from DAVID) in genes that are upregulated (red) or downregulated (blue) in only K1584R NPCs.

Supplementary Figure 5. ATRX PHDmut, K1584R, and ATRXKO differentially alter genes with function in lineage specification.

(A) Bar chart showing expression of *Klf4*, *Pou5f1 (Oct4)*, and *Nanog* as transcripts per million (TPM) in ESC and NPC states. Circles indicate individual biological replicates. Data are presented as mean values +/- SEM. * - $p < 0.05$, Welch's t-test.

(B) Bar chart showing expression of *Nestin*, *Actl6b*, and *Tubb3* as transcripts per million (TPM) in ESC and NPC states. Circles indicate individual biological replicates. Data are presented as mean values +/- SEM. * - $p < 0.05$, Welch's t-test.

(C) Heat map of expression of select mesoderm lineage genes in ESC and NPC. Each column represents the mean Z-score for three independent biological replicates from RNA-seq. Z-scores were computed across WT, PHDmut, K1584R, and ATRX KO mESCs and NPCs.

(D) Heat map of expression of select endoderm lineage genes in ESC and NPC. Each column represents the mean Z-score for three independent biological replicates from RNA-seq. Z-scores were computed across WT, PHDmut, K1584R, and ATRX KO mESCs and NPCs.

Supplementary Figure 6. ATRX PHDmut and K1584R show distinct differences in ATRX and PRC2 binding and gene expression.

(A) Pearson correlation heatmap of ATRX ChIP signal in reads per million (RPM) between WT, PHDmut, and K1584R mESCs (n = 2 replicates per cell line).

(B) Heatmap of ATRX ChIP-Seq signal across 19,796 EZH2 peaks in WT mESCs. Reads per million, RPM.

(C) Genome browser view of the *Skap2* gene showing spike-in normalized EZH2 CUT&RUN signal in WT, ATRX KD, and ATRX KO mESCs.

(D) Heatmap of spike-in normalized EZH2 CUT&RUN signal across 19,796 WT EZH2 peaks in WT and K1584R mESCs.

(E) Genomic distribution of EZH2 CUT&RUN peaks that are gained (6,029) or lost (4,429) in K1584R mESCs compared to WT.

(F) MA plot of RNA-seq expression of 13,347 genes between WT and K1584R mESCs. Red dots indicate differentially expressed genes (adjusted p-value <= 0.05, absolute log2 fold change > 1); blue dots and numbers indicate differentially expressed genes with lost promoter EZH2 in K1584R.

(G) MA plot of RNA-seq expression of 13,958 genes between WT and PHDmut mESCs. Red dots indicate differentially expressed genes (adjusted p-value <= 0.05, absolute log2 fold change > 1); blue dots and numbers indicate differentially expressed genes with Ezh2 peak at the promoter in WT.

(H) Top 5 significantly enriched processes in genes with promoters containing a gained EZH2 peak and showing reduced expression in K1584R mESCs.

Supplementary Figure 7. Distinct effects of ATRX mutations on poised enhancer-related genes.

(A) Heatmap of H3K27me3 and EZH2 CUT&RUN signal (spike-in normalized) and SUZ12 CUT&RUN (39) signal (reads per million, RPM) in WT mESCs at active, poised, and primed mESC enhancers. Enhancer numbers in each group are indicated in parentheses.

(B) Heat map of expression of 703 poised enhancer related genes in WT mESCs and NPCs. K-means clustering was used to identify 298 genes that are induced in NPCs and 405 genes that are not induced. Each column represents the mean Z-score for three independent biological replicates from RNA-seq in WT mESCs and NPCs. Z-scores were computed across WT, PHDmut, K1584R and ATRX KO mESCs and NPCs.

(C) Heatmap of expression of 295 poised enhancer related genes that are induced upon differentiation. Each column represents the mean Z-score for RNA-seq in auxin treated WT mESCs, WT NPCs, and SUZ12-AID NPCs. Z-scores were computed across three replicates of WT and SUZ12-AID mESCS and two replicates of WT and SUZ12-AID NPCs.

(D) Heatmap of expression of 298 poised enhancer related genes that are induced upon differentiation. Each column represents the mean Z-score for three independent biological replicates from RNA-seq in WT mESCs, WT NPCs, PHDmut NPCs, K1584R NPCs, and ATRX KO NPCs.

(E) Heatmap of expression of 298 poised enhancer related genes in WT and SUZ12-AID in the groups identified in Figure 5H by k-means clustering of Z scores at the NPC stage in WT, PHDmut, K1584R, and ATRX KO. Each column represents the mean Z-score for RNA-seq in auxin treated WT mESCs, WT NPCs, and SUZ12-AID NPCs. Z-scores were computed across three replicates of WT and SUZ12-AID mESCS and two replicates of WT and SUZ12-AID NPCs.

(F) Top 5 significantly enriched processes in genes from the clustered group presented on heat map in Fig 5H.

Supplementary Figure 1

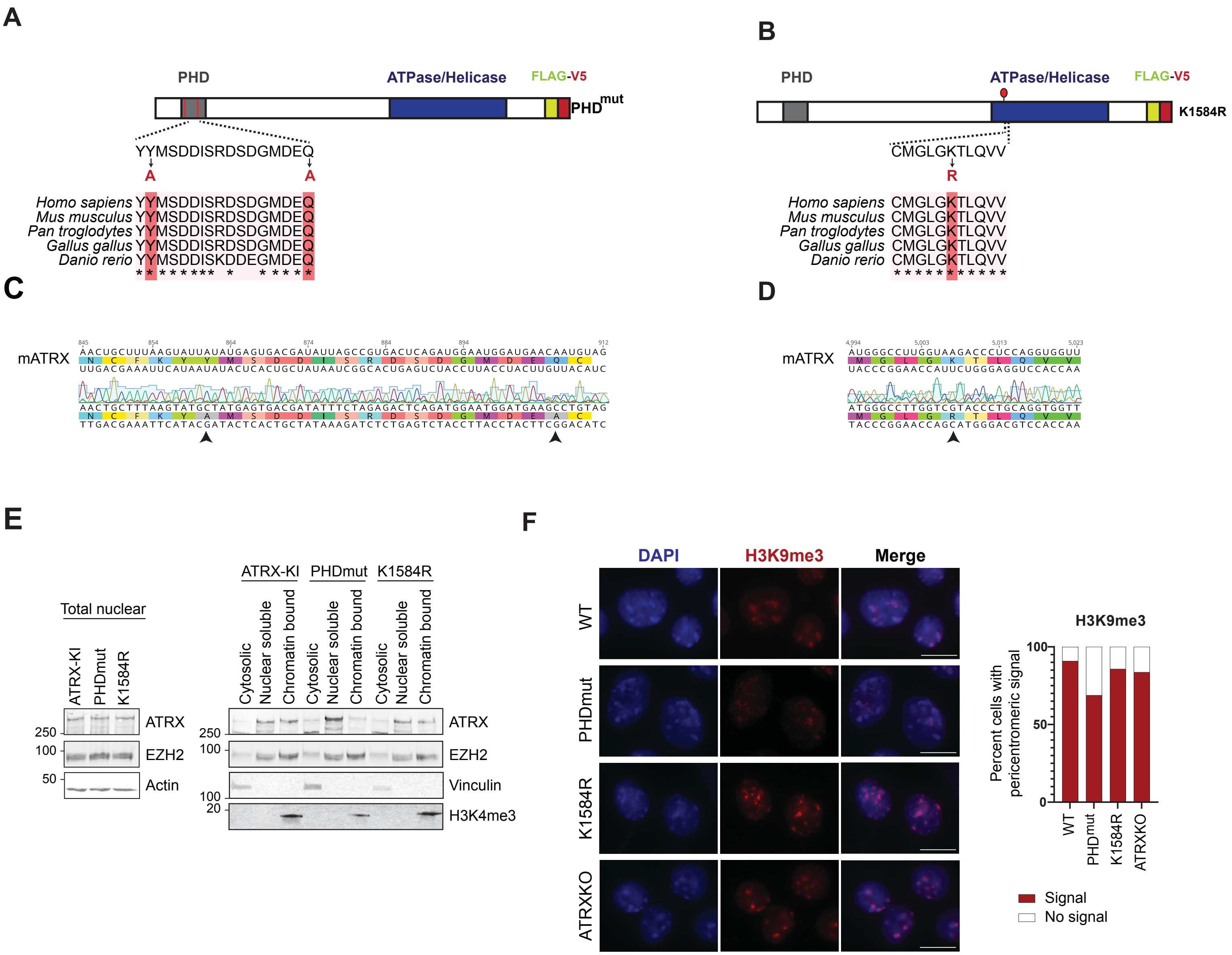

Supplementary Figure 2

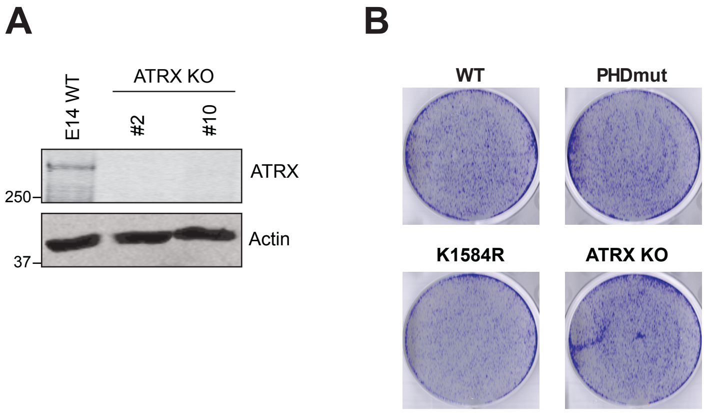

Supplementary Figure 3

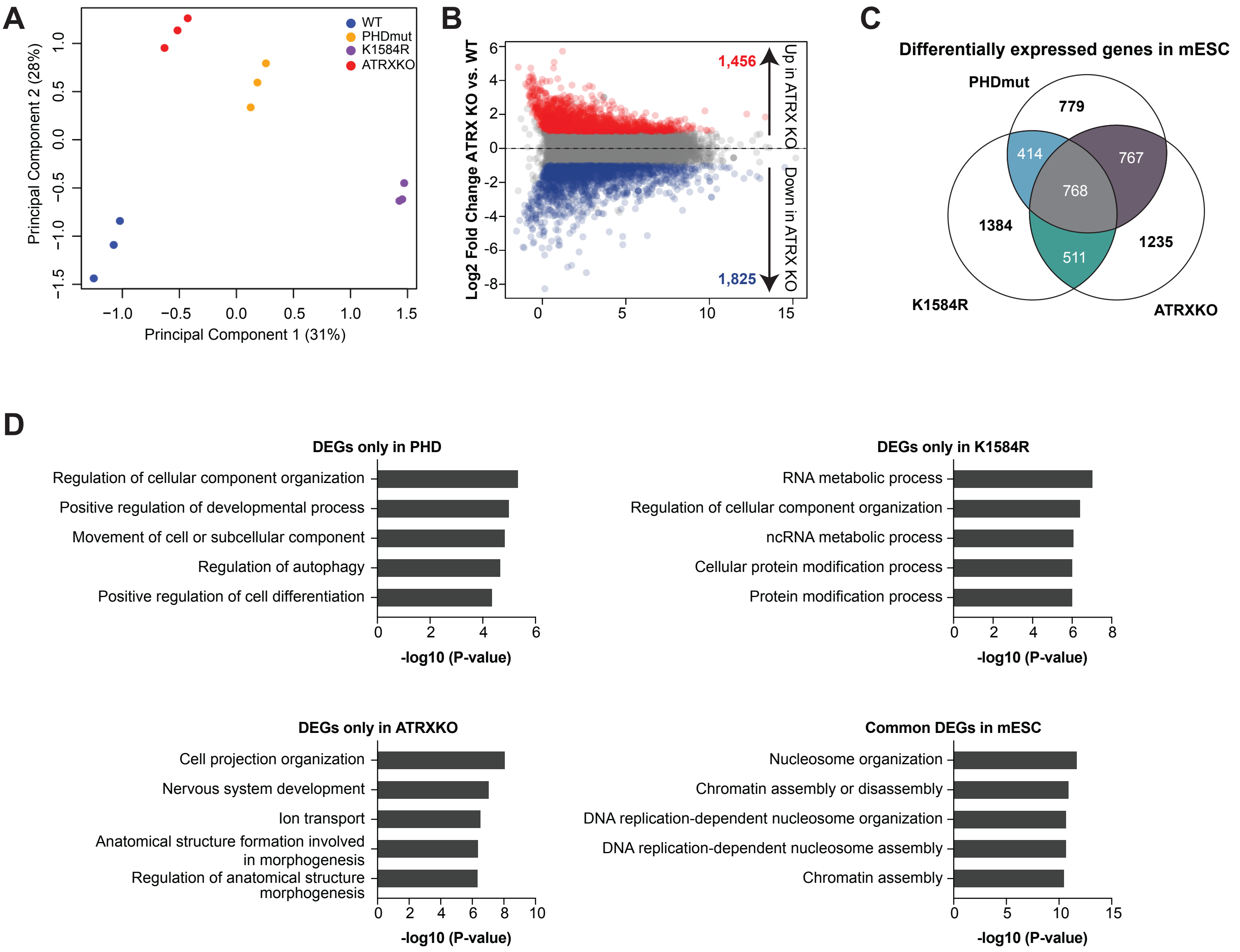

Supplementary Figure 4

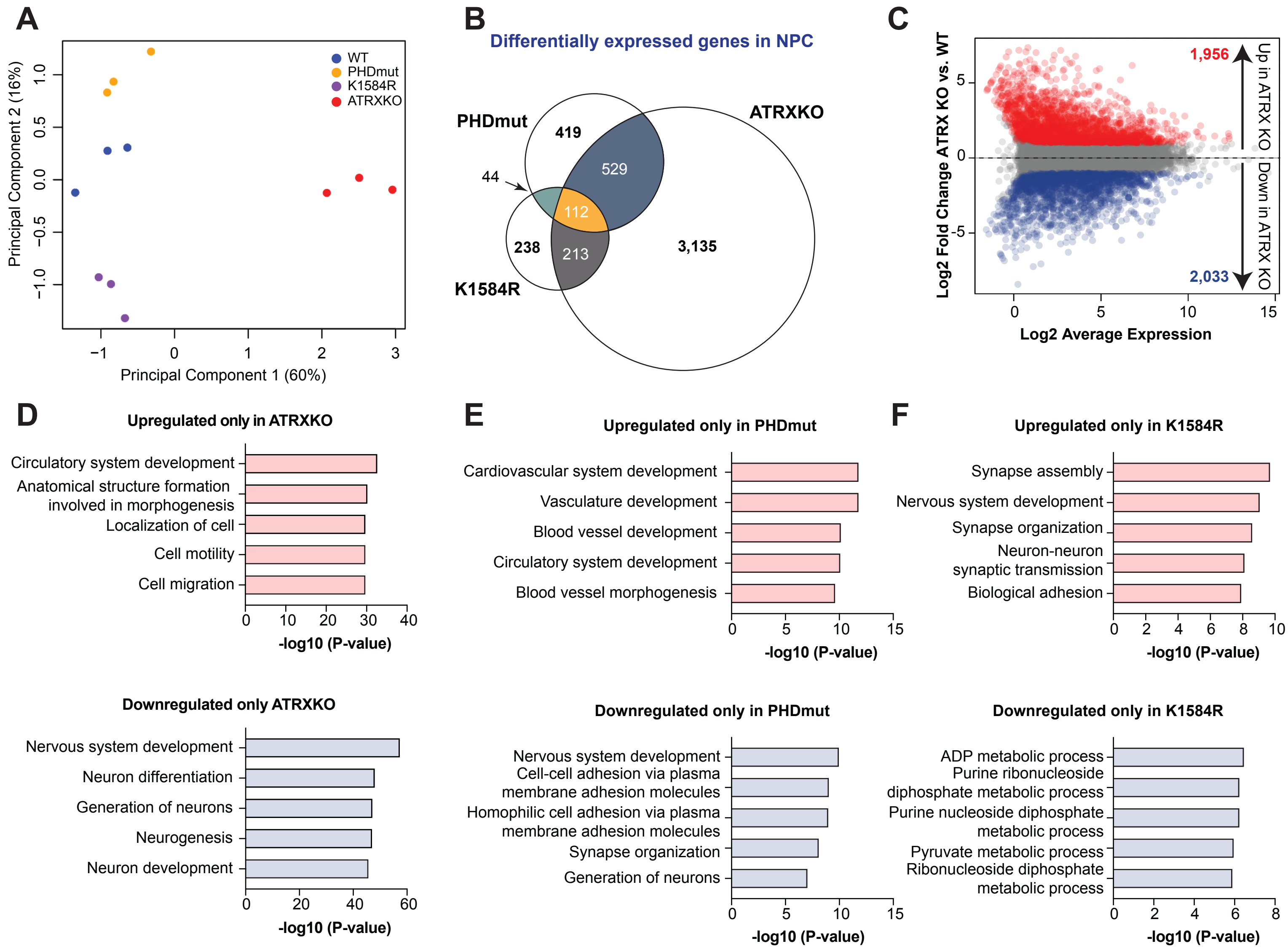

Supplementary Figure 5

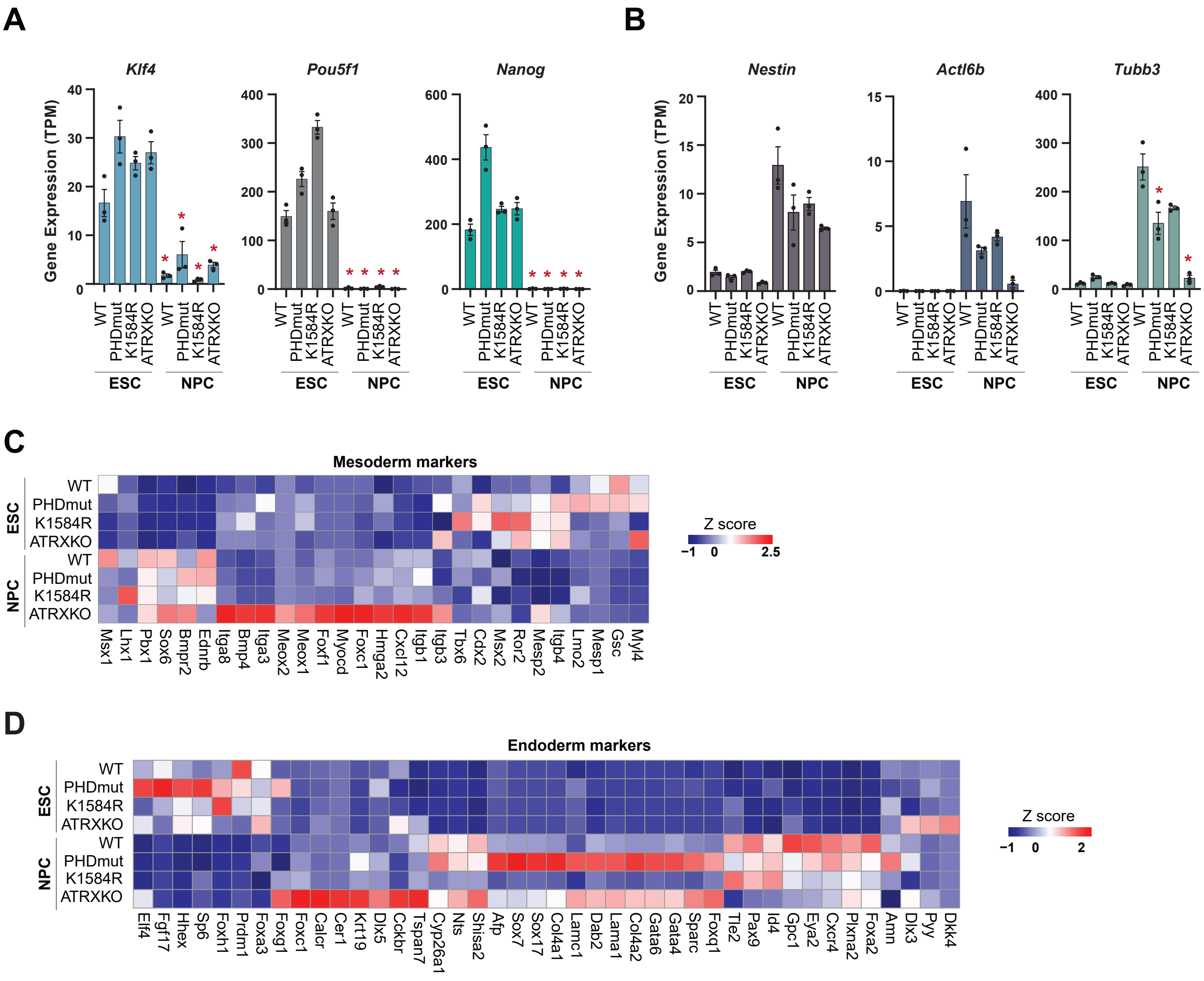

Supplementary Figure 6

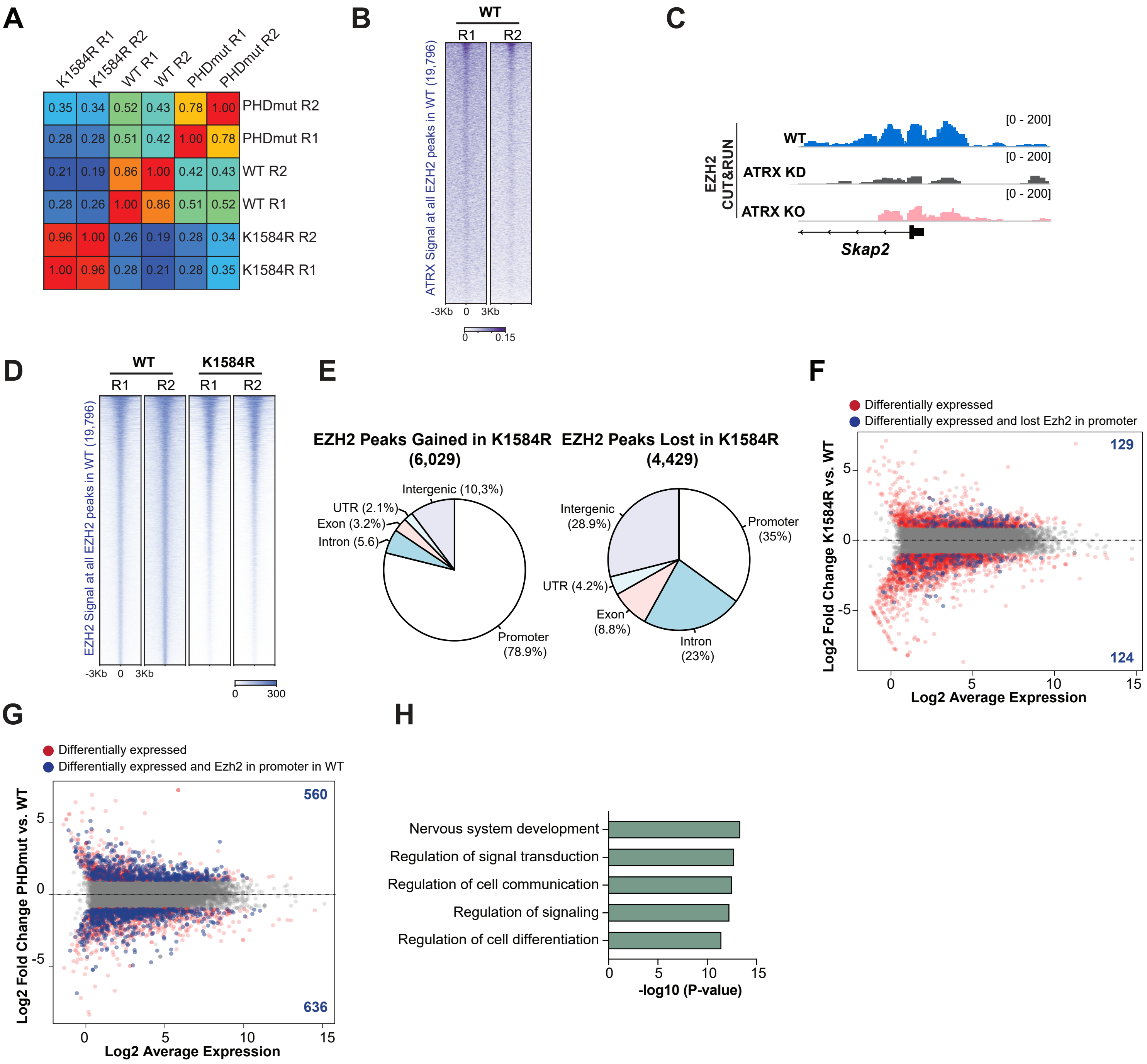

Supplementary Figure 7

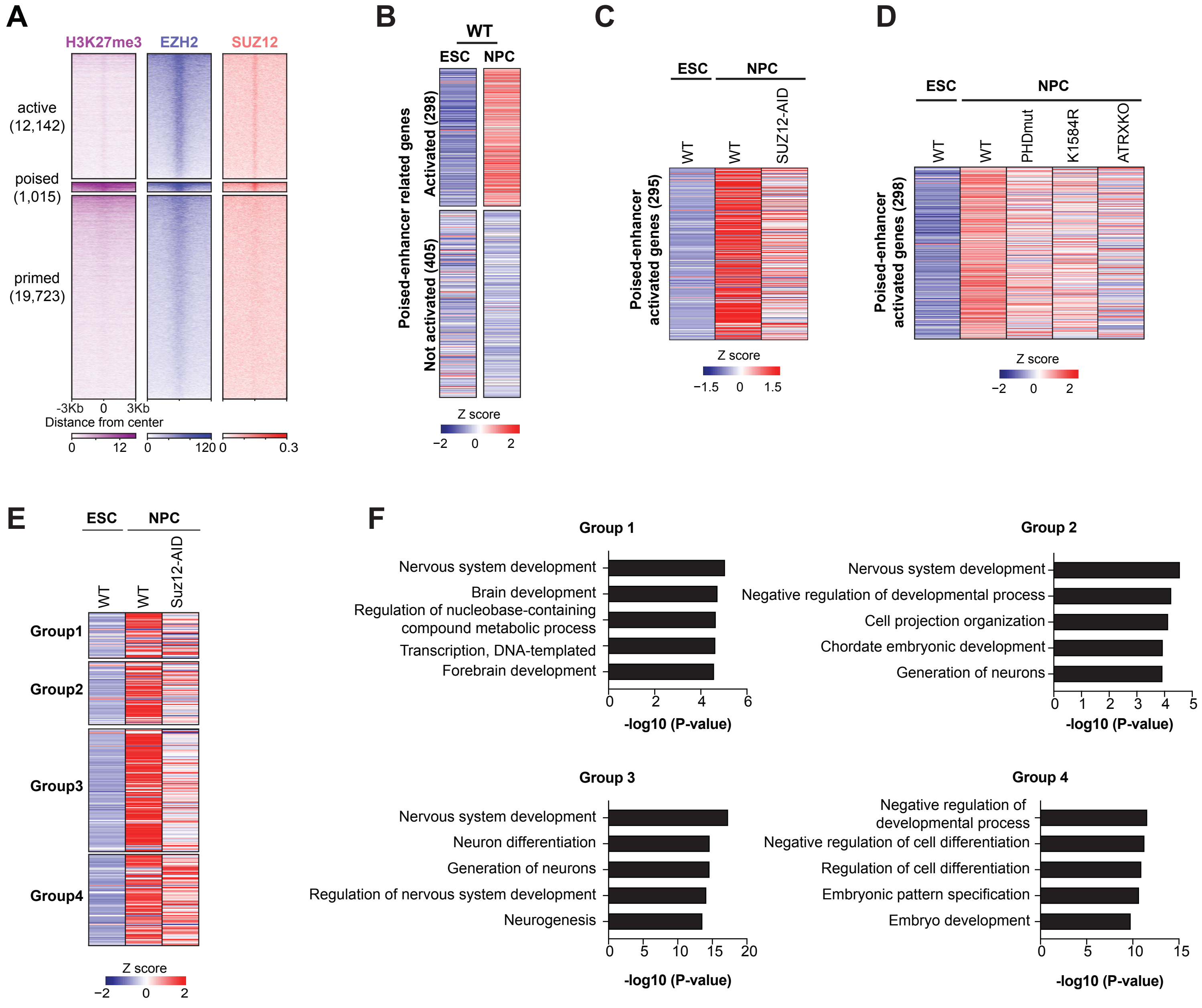

Supplement: gkac683_Supplemental_Files [file gkac683_supplemental_files.zip › Supplementary Data.pdf]
