# Supplementary material for: ATRX histone binding and helicase activities have distinct roles in neuronal differentiation
Source: Nucleic Acids Res. 2022 Aug 24;50(16):9162–74. doi: 10.1093/nar/gkac683 (PMC9458459; doi:10.1093/nar/gkac683)
Supplement: gkac683_Supplemental_Files [file gkac683_supplemental_files.zip › Supplementary Table legends.docx]

Supplementary Table 1: List of antibodies and primer sequences.

Supplementary Table 2: List of differentially expressed genes in PHDmut, K1584R, and ATRX KO vs WT in mESCs and NPCs.

Supplementary Table 3: Gene expression of poised-enhancer related genes in WT mESCs, WT NPCs, PHDmut NPCs, K1584R NPCs and ATRX KO NPCs.
